# Determination of Mycotoxin Production of *Fusarium* Species in Genetically Modified Maize Varieties by Quantitative Flow Immunocytometry

**DOI:** 10.3390/toxins9020070

**Published:** 2017-02-22

**Authors:** Hajnalka Bánáti, Béla Darvas, Szilvia Fehér-Tóth, Árpád Czéh, András Székács

**Affiliations:** 1 Agro-Environmental Research Institute, National Research and Innovation Centre, Herman Ottó út 15, H-1022 Budapest, Hungary; banati.hajnalka@naik.hu (H.B.); b.darvas@cfri.hu (B.D.); 2 Soft Flow Hungary R&D Ltd., Ürögi fasor 2/A, H-7634 Pécs, Hungary; szilvia.toth@softflow.com (S.F.-T.); arpad.czeh@softflow.com (A.C.)

**Keywords:** flow cytometry, immunoanalysis, maize, *MON 810*, *DAS-59122-7*, fumonisin B1, deoxynivalenol, zearalenone, T-2, ochratoxin A, aflatoxin B1

## Abstract

Levels of mycotoxins produced by *Fusarium* species in genetically modified (GM) and near-isogenic maize, were determined using multi-analyte, microbead-based flow immunocytometry with fluorescence detection, for the parallel quantitative determination of fumonisin B1, deoxynivalenol, zearalenone, T-2, ochratoxin A, and aflatoxin B1. Maize varieties included the genetic events *MON 810* and *DAS-59122-7*, and their isogenic counterparts. Cobs were artificially infested by *F. verticillioides* and *F. proliferatum* conidia, and contained *F. graminearum* and *F. sporotrichoides* natural infestation. The production of fumonisin B1 and deoxynivalenol was substantially affected in GM maize lines: *F. verticillioides*, with the addition of *F. graminearum* and *F. sporotrichoides*, produced significantly lower levels of fumonisin B1 (~300 mg·kg^−1^) in *DAS-59122-7* than in its isogenic line (~580 mg·kg^−1^), while *F. proliferatum*, in addition to *F. graminearum* and *F. sporotrichoides*, produced significantly higher levels of deoxynivalenol (~18 mg·kg^−1^) in *MON 810* than in its isogenic line (~5 mg·kg^−1^). *Fusarium verticillioides*, with *F. graminearum* and *F. sporotrichoides*, produced lower amounts of deoxynivalenol and zearalenone than *F. proliferatum*, with *F. graminearum* and *F. sporotrichoides*. T-2 toxin production remained unchanged when considering the maize variety. The results demonstrate the utility of the Fungi-Plex™ quantitative flow immunocytometry method, applied for the high throughput parallel determination of the target mycotoxins.

## 1. Introduction

Plant–fungal interactions and mycotoxin contamination of feed are discussed with primary interest in *Fusarium* species [1], which produce a chemically diverse array of mycotoxins: diacetoxyscirpenol, deoxynivalenol (DON), nivalenol, T-2 toxin (T-2), zearalenone (ZEA), fumonisins, fusarin C, beauvericin, moniliformin, and fusaproliferin. Fumonisins are produced by *Fusarium verticillioides* (Sacc.) Nirenberg (teleomorph: *Gibberella moniliformis* Wineland) and *F. proliferatum* (Matsush.) Nirenberg ex Gerlach & Nirenberg (teleomorph: *Gibberella intermedia* (Kuhlmann) Samuels et al.) [2]. In Europe, *F. verticillioides*, *F. graminearum* Schwabe (teleomorph: *Gibberella zeae* (Schwein.) Petch), *F. sporotrichoides* Sherbakoff (teleomorph: *Gibberella tricincta* El-Gholl et al.), and *F. subglutinans* (Wollenw. & Reinking) (teleomorh: *Gibberella fujikuroi* (Sawada) Wollenw.), are the most frequently isolated species from maize infected by plant pathogenic fungi [3]. In addition to the *Fusarium* species, *Aspergillus* spp. (aflatoxin B1—AB1 and ochratoxin A—OTA) and *Penicillium* spp. (OTA) species are also common species in maize. In our study, we used: *F. verticillioides* and *F. proliferatum*, which produce fumonisin B1 (FB1); *F. graminearum*, which usually produces DON and ZEA; and *F. sporotrichoides*, which produces T-2. Special attention has been paid to a focus on fumonisins, due to their widespread occurrence, acute toxicity, and potential carcinogenicity. The most prevalent member of the fumonisin mycotoxin class, FB1, has been classified as a Class 2B substance, i.e., probable human carcinogen [4].

Genetically modified (GM), “insect resistant” maize varieties have been suggested to affect infestation rates by *Fusarium* species, and therefore, the mycotoxin levels occurring in the commodity [5,6,7,8]. These GM maize varieties, belonging to various genetic events, produce transgenic proteins related to the δ-endotoxins (Cry toxins) of the insect’s pathogenic, Gram-positive, spore-forming *Bacillus thuringiensis* Berliner (*Bt*) bacteria, and are therefore also termed *Bt* maize varieties. The maize varieties included in this experimental series were lines of the genetic events *MON 810* and *DAS-59122-7*. In the genetic event *MON 810*, the inserted *cry1Ab* transgene produces a truncated version of the microbial Cry1Ab protoxin [9], providing protection from lepidopteran species, especially from *Ostrinia nubilalis*, and given noctuid larvae [10]. The *cry35Ab1/cry34Ab1* gene-produced binary toxin-pair have been introduced to the corn hybrid event *DAS-59122*, to provide protection from coleopteran species, especially from *Diabrotica virgifera* [11]. The hypothesized effect of these genetic events on mycotoxin production is assumed through the suppression of crop damage by insect pests (e.g., *O. nubilalis*, *Helicoverpa armigera*, etc.), that also may act as “vectors” of natural fungal infestation [12]. The occurrence of mycotoxins was also reduced in *Bt* maize, which is considered as an indirect or secondary effect of the genetic modification. Ostry et al. [13] reviewed 23 studies on different *Bt* maize events, and concluded that 19 of these experiments resulted in less contamination with fumonisins, DON, and ZEA. To ensure plant-to-plant variability, researchers often use manual insect infestations, which could lead to differences relating to larval survival. A positive correlation between the fumonisins present in maize, and the patterns of insect injury inflicted on the same plants, may be the result of diverse feeding patterns of naturally occurring insects [14]. However, differences in mycotoxin production on different Cry toxin-producing maize varieties could be independent from rates of insect damage, and could be built upon the compositional differences of the maize varieties.

Due to the complexity of possible parallel mycotoxin occurrence in biological samples, multi-analyte detection methods are of eminent importance in mycotoxin analysis. The levels of mycotoxins in maize and other agricultural commodities are commonly determined by instrumental analysis, mainly high-performance liquid chromatography coupled with tandem mass spectrometry (LC-MS/MS) [15,16,17,18], providing quantitative results with high sensitivity and specificity. One of the advantages of sensitive coupled mass spectrometric analysis is that, as a multi-residue method, it is capable of determining numerous mycotoxin metabolites along the parent compounds [19], and in turn, LC-MS/MS became the reference method for mycotoxin analysis. In addition, immunoanalytical methods, including enzyme-linked immunosorbent assays (ELISAs), are widely used for mycotoxin analysis [20,21,22] and other immunoanalytical techniques, including immunosensorics [23,24,25,26,27,28] and flow immunocytometry [29,30,31], which are on the rise. These advanced methods utilize biochemical molecular recognition elements (e.g., antibodies, aptamers, etc.) for the specific detection of given mycotoxins, and the use of numerous molecular recognition elements with varying specificity allows the parallel determination of several mycotoxins. Fungi-Plex™, a flow cytometry-based multiplexed competitive assay using fluorescent microbeads, has been developed for the simultaneous detection of six major mycotoxins from a single sample extraction [32].

The objective of the present study was to assess the utility of the novel immunoanalytical method, quantitative flow immunocytometry, for the high throughput multi-analyte determination of six mycotoxins, including FB1, DON, ZEA, and T-2, produced by *Fusarium* species, as well as AB1 and OTA, produced by *Aspergillus* and *Penicillium* species, respectively. To characterize the possible interactions between different Cry toxins originating from *Bt* maize, and mycotoxins originating from *Fusarium* species, levels of these mycotoxins were analyzed in *Bt* maize varieties and corresponding isogenic lines.

## 2. Results

### 2.1. Flow Cytometry by the Fungi-Plex*™* Kit

**Figure 1:**
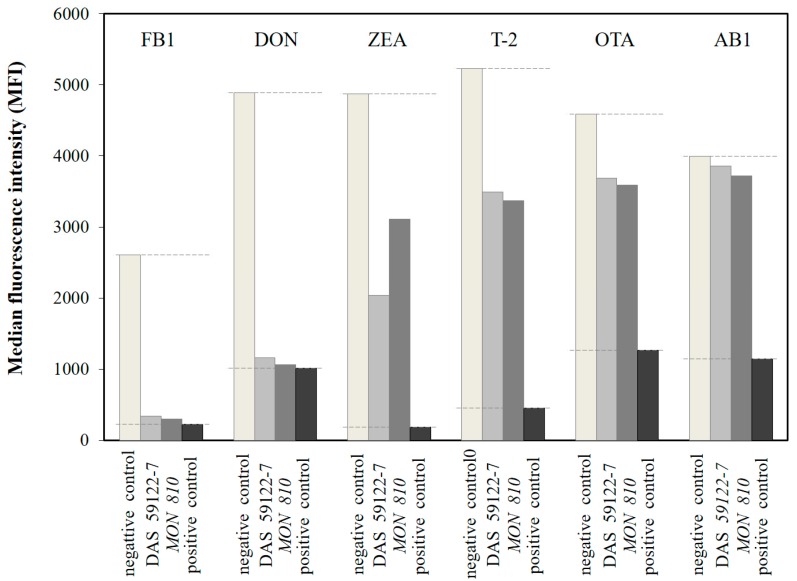
Histogram of median fluorescence intensities (MFIs) detected in the Fungi-Plex™ multiplex flow cytometric determination for negative controls (white columns) and positive controls (black columns), as well as for the typical values of maize, of genetic events *DAS 59122-7* (light grey columns) and *MON 810* (dark grey columns) for mycotoxins fumonisin B1 (FB1), deoxynivalenol (DON), zearalenone (ZEA), T-2 toxin (T-2), ochratoxin A (OTA), and aflatoxin B1 (AB1).

Measurement with the validated Fungi-Plex™ kit [32] realizes mycotoxin determination in a competitive assay format, using the Lab on Beads^TM^ technology. An immunoreaction between mycotoxin molecules and their immobilized specific antibodies takes place on the surface of polymeric microbeads, which are internally dyed with fluorophores, providing several levels of fluorescent intensities which permit possible clusterization [33]. The measurement is based on the competition between the mycotoxin molecules and the mycotoxin-coupled phycoerythrin (PE) macromolecules: the concentration of the mycotoxin-PE conjugates supplied by the assay kit, is inversely proportional to the mycotoxin concentration in the analyzed sample. To allow multiplex parallel mycotoxin determination, separate bead populations (capture beads) with different fluorescence characteristics, and coated with specific antibodies prepared against the target analytes, are used in parallel, and combined fluorescent signals are processed by advanced data analysis [34]. In this case, six bead populations with specific antibodies anchored to the mycotoxins AB1, ZEA, OTA, FB1, DON, and T-2 toxin, were supplied. The bead populations are capture-bead types of discrete median fluorescence intensity (MFI), at 670 nm. As the beads pass through the detection cuvette of the flow cytometer, signal intensities are detected at 580 nm of the PE fluorescence for the actual assay inhibition signal (corresponding to the concentration of the given mycotoxin), and at 640 nm to identify the clustering fluorescence, i.e., the bead type corresponding to the given mycotoxin. MFIs were determined from three replicates of each sample, including all of the maize samples (GM or isogenic), as well as the negative and positive controls. Due to the differing affinities of the antibodies, different operating MFI ranges were observed for different mycotoxins, i.e., 224–2666, 1014–4893, 185–4870, 449–5233, 1264–4593, and 1139–3995 for the mycotoxins FB1, DON, ZEA, T-2, OTA, and AB1, respectively. Typical MFI values for GM maize varieties, as well as for the negative and positive controls obtained for each mycotoxin, are depicted in Figure 1.

Using the above determination system, sigmoid standard curves were obtained for each mycotoxin analyzed in parallel, at individual concentration ranges depending on the antibody’s affinity characteristics. The main analytical parameters, including the analyte concentration corresponding to the inflection point of the sigmoid curve (C_50_), the limit of detection (LOD, defined as the analyte concentration corresponding to an assay signal of the blank signal (upper plateau) minus three standard deviations of the blank), the slope of the standard curve at the inflection point, and the regression coefficient (*R*^2^) of the non-linear (sigmoid) curve fitting, are listed in Table 1.

The standard curves of OTA, AB1, and ZEA run quite close to each other, and allow analyte determination at the lowest concentration range (approx. 10–3000 pg·mL^−1^). In contrast, DON and FB1 are detected at the highest concentration range (approx. 800–10,000 pg·mL^−1^), and the standard curve for T-2 runs in between. These are well reflected in both the C_50_ and LOD values in the assay solution. The LOD values for the determination expressed as mg mycotoxin per kg maize commodity, was calculated with the sample preparation step used in the present study. Although the concentration ranges of the quasi-linear parts of the standard curves for given mycotoxins, may differ from each other by two orders of magnitude, all of the curves are similar in shape, as can be seen by their almost uniform slopes and excellent regression. Slopes at the inflexion point (C_50_) were found to range from −0.99 to −0.79. This is an excellent feature for analytical sensitivity (concentration dependence of the signal), as slopes near ׀1׀ are considered ideal. The excellent regression of the non-linear curve fit is seen in the regression coefficients (*R*^2^), which are all above 0.9988.

The reproducibility of the Fungi-Plex™ kit was determined to be sufficient for routine use [32]. Thus, within a day, (intra-assay) variabilities near the C_50_ value in the assay were found to be 7.4%, 7.6%, 8.0%, 7.9%, 6.0%, and 6.2%, for AB1, ZEA, OTA, T-2, DON, and FB1, respectively. Corresponding day-to-day (inter-assay) variabilities were 12.3%, 12.1%, 9.1%, 8.5%, 10.2%, and 10.9%, respectively.

Cross-reactivities (CRs) of the specific antibodies to structurally-related mycotoxins or metabolites, defined as the percentage obtained by calculating the ratio of the C_50_ value of the reference mycotoxin to that of the given mycotoxin derivative, were determined in the corresponding immunoanalytical method. Thus, the AB1-specific antibody showed CR to aflatoxin B2 (76%), aflatoxin M1 (79%), aflatoxin M2 (33%), aflatoxin G1 (55%), and aflatoxin G2 (6%); the ZEA-specific antibody to zearalanone (138%), α-zearalenol (91%), β-zearalenol (21%), α-zearalanol (69%), and β-zearalanol (6%); the FB1-specific antibody to fumonisin B2 (91.1%) and fumonisin B3 (209%); the T-2-specific antibody to acetyl-T-2 (12.3%), 4-deacetyl-T-2 (3.4%), and iso-T-2 (2.5%); the DON-specific antibody to 3- and 15-acetyl-DON (>80%); and the OTA-specific antibody to ochratoxin B (9.3%). In contrast, no observable CRs were recorded for the six types of antibodies with mycotoxins belonging to different classes than their target analytes. These CR patterns indicate that the T-2- and OTA-specific antibodies are specific to their target analytes, while the other anitbodies show significant CRs with related mycotoxins or metabolites. Nonetheless, the ZEA-specific antibodies can also be considered target-specific, as ZEA is known to be the major mycotoxin produced by several *Fusarium* species, including *F. graminearum*, and the related compounds listed above are either semi-synthetic derivatives, or those produced during its metabolism. The same can be stated about DON, as its acetylated derivatives listed above are known to be formed at low levels, compared to DON. As the FB1-specific antibody shows high CR to fumonisin B2 and B3, the immunoanalytical method can be claimed to be capable of detecting fumonisins (not necessarily FB1 alone). The method based on AB1-specific antibodies can be considered aflatoxin-specific, but other classes of aflatoxins (aflatoxins M or G) are known to be produced under different physiological conditions than aflatoxins B; moreover, aflatoxins were not detected in the current study.

### 2.2. Mycotoxin Levels in Corn Ear Cross Disks

**Figure 2:**
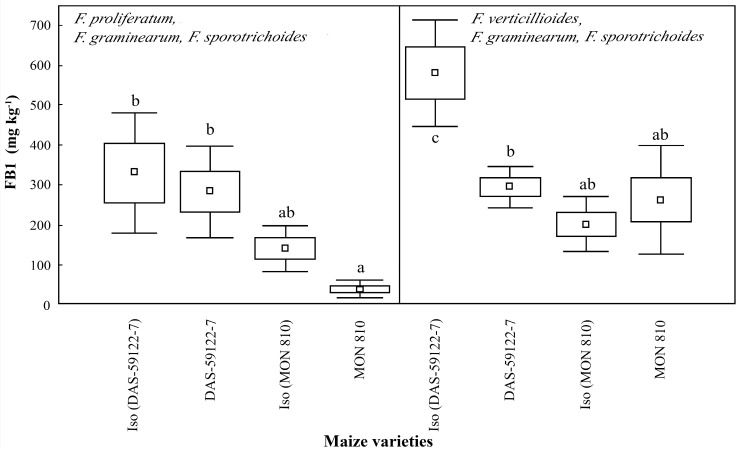
Fumonisin B1 (FB1) content (mg·kg^−1^) in maize ear cobs of GM and non-GM maize varieties infested by *Fusarium* species (*F. proliferatum*—artificial infestation, *F. graminearum* and *F. sporotrichoides*—natural background infestation and *F. verticillioides—*artificial infestation, *F. graminearum* and *F. sporotrichoides*—natural background infestation). Different alphabetical letters (a, b, ab) indicate statistical differences, *p* = 0.5.

FB1 was the major mycotoxin present in all samples [35]. In the case of *F. proliferatum*, with *F. graminearum* and *F. sporotrichoides*, there were no significant differences within the maize line pairs. *F. proliferatum*, with *F. graminearum* and *F. sporotrichoides*, produced the lowest levels of FB1 on ear cobs containing the Cry1Ab toxin (*MON 810*), but the difference was not significant when compared to the near-isogenic line. In the isogenic line of *DAS-59122-7*, *F. verticillioides*, with *F. graminearum* and *F. sporotrichoides*, produced significantly higher levels of FB1 (~580 mg·kg^−1^) than on the ear cobs of *DAS-59122-7 Bt* (~300 mg·kg^−1^). On the *DAS-59122-7* ear cobs, both *Fusarium* species complexes produced more (300–600 mg·kg^−1^) FB1 than on the *MON 810* lines (50–250 mg·kg^−1^) (Figure 2). This difference suggests that not only the Cry toxin content, but also the difference in the composition of the corn ears, appear to be important factors in mycotoxin production. These measurements suggest that *Fusarium* species growing on maize containing Cry toxins, may produce the same level of FBI as, or less than, their isogenic varieties. The effect appears to depend both on the type of the Cry toxin, and the different mycotoxin-producing species. The results are in agreement with the decreased, but not significantly reduced, fumonisin content reported in Cry1Ab-producing *Bt* maize, when compared to non-*Bt* lines [36,37].

**Figure 3:**
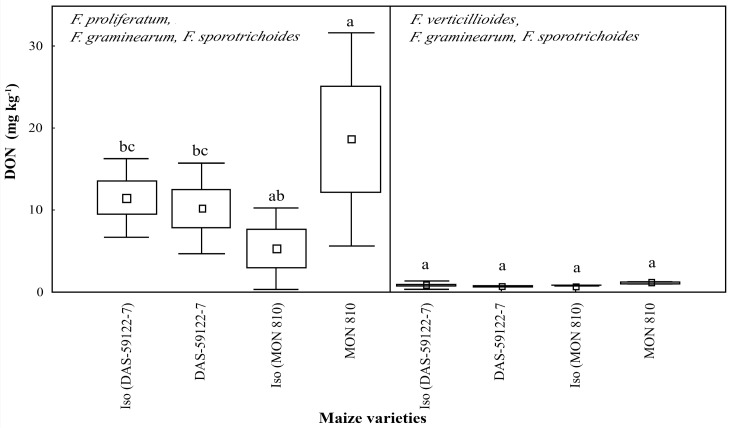
Deoxynivalenol (DON) content (mg·kg^−1^) in maize ear cob sample cobs of GM and non-GM maize varieties infested by *Fusarium* species (*F. proliferatum*—artificial infestation, *F. graminearum* and *F. sporotrichoides*—natural background infestation and *F. verticillioides*—artificial infestation, *F. graminearum* and *F. sporotrichoides*—natural background infestation). Different alphabetical letters (a, ab, bc) indicate statistical differences, *p* = 0.5.

*Fusarium proliferatum*, with *F. graminearum* and *F. sporotrichoides*, produced an average of 10–20 mg·kg^−1^ of DON [3,35,38]. In *MON 810* ear cobs containing the Cry1Ab toxin, the level of DON was significantly higher (~18 mg·kg^−1^), when compared to the isogenic line (~5 mg·kg^−1^) (Figure 3). It seems that DON production is enhanced in the maize ear cob containing the Cry1Ab toxin. Other experiments showed decreased concentrations of fumonisins and ZEA, but the level of DON was found to be slightly increased in *MON 810* maize [8]. This tendency of an increased DON level is not explained by the insect damage rate modified by the genetic event, and could rather be attributed to the compositional differences of the GM maize variety.

**Figure 4:**
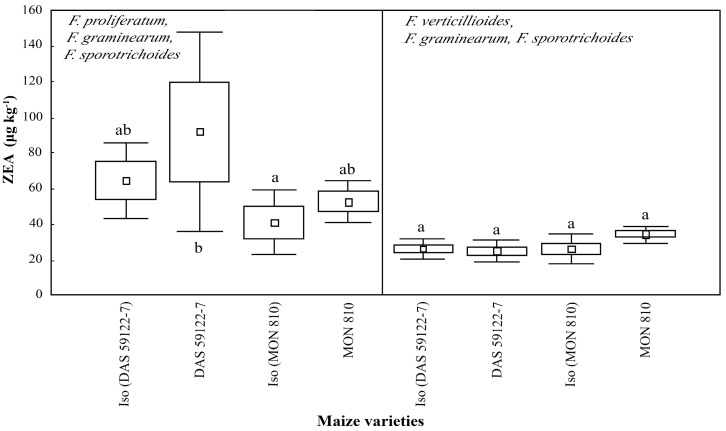
Zearalenone (ZEA) content (μg·kg^−1^) in maize ear cobs of GM and non-GM maize varieties infested by *Fusarium* species (*F. proliferatum*—artificial infestation, *F. graminearum* and *F. sporotrichoides*—natural background infestation and *F. verticillioides*—artificial infestation, *F. graminearum* and *F. sporotrichoides*—natural background infestation). Different alphabetical letters (a, b, ab) indicate statistical differences, *p* = 0.5.

ZEA production was found to be low [3,35]: *F. proliferatum*, with *F. graminearum* and *F. sporotrichoides*, produced ~50–100 μg·kg^−1^, while *F. verticillioides*, with *F. graminearum* and *F. sporotrichoides*, produced ~20–30 μg·kg^−1^ (Figure 4). In both GM events (*MON 810* and *DAS-59122-7*), the amount of ZEA increased slightly, when compared to that in their isogenic lines, but there were no obvious statistically significant differences.

*Fusarium verticillioides*, with *F. graminearum* and *F. sporotrichoides*, produce lower amounts of DON and ZEA than *F. proliferatum* with *F. graminearum* and *F. sporotrichoides*. The results indicate that the biosynthesis of different mycotoxins by the *Fusarium* spp. may be affected by interspecific interactions. *Fusarium graminearum* is the source of DON and ZEA, and *F. sporotrichoides* is the source of T-2.

**Figure 5:**
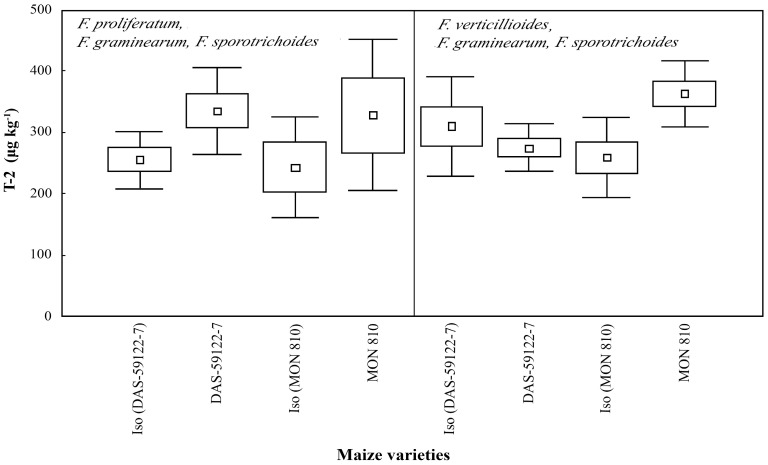
T-2 toxin content (μg·kg^−1^) in maize ear cobs of GM and non-GM maize varieties infested by *Fusarium* species (*F. proliferatum*—artificial infestation, *F. graminearum* and *F. sporotrichoides*—natural background infestation and *F. verticillioides*—artificial infestation, *F. graminearum* and *F. sporotrichoides*—natural background infestation).

Both *Fusarium* species complexes produced T-2 toxin (250–350 μg·kg^−1^) (Figure 5). Differences in T-2 production have not been found [3,38], neither among different genetic events compared to their near-isogenic lines, nor amongst different *Fusarium* species. In the present study, we did not find any interspecific interaction based on T-2 production.

As expected, AFB1 and OTA were not found in the corn samples, as *F. proliferatum*, *F. graminearum*, *F. verticillioides*, and *F. sporotrichoides* are known not to produce these mycotoxins [39,40], but our measurements (data not shown) confirm that no AB1- and OTA-producing *Aspergillus* or *Penicillium* species occurred in this experimental series.

## 3. Discussion

As indicated by our earlier field experiments, *Helicoverpa armigera* (Hübner) (Lep., Noctuidae) larvae caused mostly apical corn ear damage, and only 20%–30% of the larval damage was followed by a *Fusarium* spp. infection [9]. As a consequence, only a small part of *Fusarium* spp. infection cases may be connected to insect larval damage.

*Bacillus thuringiensis* strains are ubiquitously present in our environment, as soil-borne bacteria, and simultaneously, as insect larval pathogens. The identification of the protein composition in the parasporal bodies of numerous strains, and the discovery of the unique physico-chemical features and biological specificity of the Cry toxin proteins, has led to several landmark events in pest control practices [41]. For example, Cry1 toxin groups have lethal effects on caterpillars, while Cry3 toxin groups have the same effects on some beetle larvae. Soil-borne bacteria usually have unique strategies for surviving in the soil, including sporulation and the production of toxic secondary metabolites (allelochemicals) that may modify the colonization of neighboring microorganisms [42]. Crop residues are the source of carbon in soil, and changes in their nutritional quality may also modify the activity of these organisms [9,43]. Differences in the composition of *Bt* plants and near-isogenic counterparts, may cause changes in their suitability as a food source for *Fusarium* species, and their interspecific interaction may alter their mycotoxin production. Cry toxin levels in the corn ears are significantly different in the two *Bt* corn varieties which were examined. The Cry1Ab toxin level in *MON 810* dry corn grain, is 0.63 ± 0.06 ng·mg^−1^ [10], while that of Cry34Ab1/Cry35Ab1 unique binary toxins in *DAS-59122-7*, is 50 ± 16 and 1 ± 0.3 ng·mg^−1^, respectively [11]. Finally, chemotypes also exist among *Fusarium* species, making mycotoxin production variable, even within a single species [44].

## 4. Conclusions

The Fungi-Plex™ multiplex flow immunocytometric mycotoxin assay kit, utilizing the Lab on Beads technology, was proven to be applicable for the quantitative determination of six mycotoxins (AB1, ZEA, OTA, FB1, DON, and T-2) in GM maize varieties of the genetic events *MON 810* and *DAS-59122-7*, as well as their near-isogenic lines, with excellent standard sigmoid inhibition curve regression characteristics and LODs between 0.005 (AB1) and 0.406 mg·kg^−1^ (DON). Consequently, the concentration ranges of the linear parts of the standard curves for the mycotoxins, ranged within two orders of magnitude, and the slopes were between −0.79 to −0.99.

The present study indicated that fumonisin production by *F. verticillioides*, with *F. graminearum* and *F. sporotrichoides*, was changed during *MON 810* and *DAS-59122-7* events (Figure 2), relative to their near-isogenic lines, while DON production by *F. proliferatum*, with *F. graminearum* and *F. sporotrichoides*, was changed in the opposite manner (Figure 3). A significant reduction of FB1 levels in *DAS-59122-7* by *F. verticillioides* with *F. gramiearum* and *F. sporotrichoides*, but not in the case of *F. proliferatum* with *F. graminearum* and *F. sporotrichoides*, was revealed. In contrast, the level of DON was significantly higher in *MON 810* than in its near-isogenic line by *F. proliferatum* with *F. graminearum* and *F. sporotrichoides*, but no differences were found in the case of *F. verticillioides* with *F. graminearum* and *F. sporotrichoides*. The findings have long-ranging implications not only to the *Bt* maize lines studied here, but also to other GM maize varieties, particularly of stacked events, where insect resistance is combined with herbicide tolerance. Pesticide applications are known to influence mycotoxin production in maize, and the herbicide active ingredient glyphosate along with its formulating agent polyethoxylated tallowamine is being increasingly applied on glyphosate-resistant GM crops. A further study may be required to divide the effects of each factor on the modified mycotoxin production, including variable composition of the corn ears as biological matrices related to the breeding line, Cry toxin types and the production intensity in *Bt* events, and the chemotypes of *Fusarium* species.

## 5. Materials and Methods

### 5.1. Materials

Acetonitrile as an organic solvent for sample extraction was purchased from Reanal (Reanal Fine Chemicals Corp., Budapest, Hungary). All other chemicals were purchased from Sigma-Aldrich (Sigma-Aldrich Co. LLC, Budapest, Hungary). The *F. proliferatum* strain was isolated from corn field samples (Páty) [9], identified by Árpád Szécsi (Plant Protection Institute, Centre for Agricultural Research, Hungarian Academy of Sciences, Budapest, Hungary), cultured under laboratory conditions, and used for artificial inoculation. For inoculation with the *F. verticillioides* strain, the F146 isolate used, originated from Árpád Szécsi. After the incubation period, we isolated it from the mycelia *F. graminearum* and *F. sporotrichoides*, resulting in field (Julianna-major) cob infestation without any visible symptoms at the collection time. We did not use any sterilization process on the natural cob disks, but checked the *Fusarium* conidia.

### 5.2. Instrumentation and Methods

The quantitative toxin content determination of the samples was carried out using the Fungi-Plex™ multiplex mycotoxin assay kit (Soft Flow Hungary Ltd., Pécs, Hungary), a microbead-based, flow cytometric analytical assay, developed for the qualitative and quantitative detection of mycotoxins AB1, ZEA, OTA, FB1, DON, and T-2 [45]. For the analysis, a BD FACSArray^TM^ Bioanalyzer (BD Biosciences, Franklin Lakes, NJ, USA) was used [32].

### 5.3. Maize Samples and Inoculation

Two different varieties of *Bt* maize lines (*MON 810* and *DAS-59122-7*), and two corresponding near isogenic maize lines, were involved in the experiment. Maize was cultivated at Julianna-major, Nagykovácsi (Hungary). No insecticide, herbicide, or fungicide was applied during the cultivation period. Corn ears were covered with paper bags before the beginning of the silking period. Pollen was also collected in paper bags. *MON 810 × MON 810* and *DAS-59122-7 × DAS-59122-7* crossings were made by hand pollination, to protect the genetic material of the pollen. Samples of the corn ears (five repetitions of both maize varieties) were taken at the R4 phenological stage from maize plants grown at field conditions, and 2.5 cm thick cross disks of a uniform diameter were cut from the middle section of the corn ears.

*Fusarium* suspensions for artificial infestation were prepared from neat cultures of *Fusarium* strains (*F. proliferatum* and *F. verticillioides*), at an ~80.000 per mm^3^ microconidium density, in distilled water containing 0.1% Tween 20, and the suspensions were stirred at room temperature for 1 hr. Corn ear cross disks (28–30 pieces originated from 5-5 cobs) were fully immersed in the *Fusarium* suspension for 30 s, and were subsequently incubated at 25 °C for up to three weeks, until ~70%–95% of the cross disk surface was covered with white mycelia. To avoid cross-contamination, each cross disk was handled in individual containers closed with a lid, resulting in high relative humidity. Disks with any incidental—limited to one seed—grey (<30% *Aspergillus* sp.) or green (<7% *Penicillium* sp.) mycelia, were discarded from the experiment. No more than a week was enough for 40%–60% surface colonization of each corn ear cross disk by *Fusarium* spp. mycelia. After examining the measured white mycelia, we recognized that the cobs were naturally infested with *F. graminearum* and *F. sporotrichoides* in the field. Therefore, our results represent the mycotoxin production of three *Fusarium* species under the same duration. After three weeks of incubation, cob disks were individually stored at −50 °C, before mycotoxins measurements were taken.

### 5.4. Sample Preparation

Only *Fusarium* mycelium samples (approximately 1 g) of the corn ear cross discs were cut and homogenized in a mortar. A total of 50 mg of the homogenate was transferred into Eppendorf tubes, 1 mL of 84% aqueous acetonitrile was added, and the samples were vortexed for 2 min, shaken on a Titramax 101 orbital vibrating platform shaker (Heidolf Instruments GmbH & Co., Schwabach, Germany) for 10 mins, before being centrifuged at 12.000 rpm for 10 min, at 4 °C.

### 5.5. Determination of Mycotoxins by Flow Cytometry

Measurements were performed on the extracted samples, diluted 1:25, 1:100, and 1:500 in the kit standard diluent buffer, according to the modified Application Protocol for the Fungi-Plex™ kit (Soft Flow Hungary Ltd., Pécs, Hungary, 2010). Analytical standards of the determined mycotoxins were applied in serial dilution series at eight concentrations, within individual concentration ranges for each mycotoxin, between 0 and 100,000 pg·mL^−1^. Aliquots (100 μL) of the diluted samples or the standard solutions were pipetted onto 96-well microplates with filter bottom wells, and 50 μL of the kit’s detection reagent mix was added to each well (with direct exposure to light avoided), 50 μL of the freshly vortexed kit’s capture bead mix was added to each well, and the microplate was incubated at room temperature for 45 min, with continuous orbital shaking at 650 rpm. Upon completion, a gentle vacuum (below 40 kPa) was applied to the bottom of the filter microplate, and the content of the wells were drained. The wells were then washed three times with 200 μL of the kit’s wash buffer, with the washing liquid similarly drained each time. Then, the content of each well was resuspended in 200 μL of the kit’s wash buffer, by shaking the microplate on the orbital shaker at 600 rpm, and the homogenized bead suspensions were measured in the flow cytometer.

### 5.6. Statistical Analysis

Following flow cytometric determination, the acquired data (FCS list mode sample data) were analyzed using the FCAP Array™ v3.0 Analysis Software (BD Biosciences, Franklin Lakes, NJ, USA), for clustering the acquired bead populations, and for processing the clustered reporter fluorescence data. For statistical analysis, the 500-fold dilution was selected for FB1, and in the case of DON, ZEA, and T-2, the 25-fold sample dilution was chosen. The normality of the data distribution was evaluated by the Shapiro–Wilk’s test. Experimental data sets showed a normal distribution. Data were tested at a 95% confidence. Data were subjected to one-way analysis of variance (ANOVA), and a post hoc Tukey test was carried out. Statistical analyses were performed using the Statistica software (StatSoft, Inc., Tulsa, OK, USA).

## Figures and Tables

**Table 1:** Analytical parameters of the standard sigmoid inhibition curves obtained in the Fungi-Plex™ flow cytometric determination of six mycotoxins in buffer.

Mycotoxin	C_50_^a^ (ng·mL^−1^)	Slope ^b^	*R*^2 c^	LOD ^d^ (mg·kg^−1^)
aflatoxin B1 (AB1)	0.145 ± 0.003	−0.79	99.97	0.005
zearalenone (ZEA)	0.185 ± 0.001	−0.86	99.92	0.009
ochratoxin A (OTA)	0.175 ± 0.004	−0.93	99.88	0.006
T-2 toxin (T-2)	1.667 ± 0.024	−0.91	99.98	0.076
deoxynivalenol (DON)	3.107 ± 0.026	−0.92	99.89	0.406
fumonisin B1 (FB1)	4.926 ± 0.097	−0.99	99.90	0.116

^a^ Analyte concentration corresponding to the inflection point (50%) of the sigmoid curve; ^b^ Steepness of the sigmoid calibration curve at the C_50_ point; ^c^ Regression coefficient of the non-linear (sigmoid) curve fit; ^d^ Limit of detection (LOD), overall value (mg mycotoxin per kg maize) calculated with sample preparation parameters considered at minimal applied dilution (1:25). LOD is defined as the analyte concentration corresponding to an assay signal of the blank signal minus three standard deviations of the blank.

## References

[1] Logrieco A., Bottalico A., Mulé G., Moretti A., Perrone G. (2003). Epidemiology of toxigenic fungi and their associated mycotoxins for some Mediterranean crops. Eur. J. Plant Pathol..

[2] White D.G. (1999). Compendium of Corn Diseases.

[3] Eckard S., Wettstein F.E., Forrer H.-R., Vogelgsang S. (2011). Incidence of *Fusarium* species and mycotoxins in silage maize. Toxins.

[4] International Agency for Research on Cancer (IARC) (1993). Monographs on the Evaluation of Carcinogenic Risks to Humans.

[5] Duvick J. (2001). Prospects for reducing fumonisin contamination of maize through genetic modification. Environ. Health Perspect..

[6] Schaafsma A.W., Hooker D.C., Baute T.S., Illincic-Tamburic L. (2002). Effect of *Bt*-corn hybrids on deoxynivalenol content in grain at harvest. Plant Dis..

[7] Clements M.J., Campbell K.W., Maragos C.M., Pilcher C., Headrick J.M., Pataky J.K., White D.G. (2003). Influence of Cry1Ab protein and hybrid genotype on fumonisin contamination and *Fusarium* ear rot of corn. Crop Sci..

[8] Folcher L., Delos M., Marengue E., Jarry M., Weissenberger A., Eychenne N., Regnault-Roger C. (2010). Lower mycotoxin levels in *Bt* maize grain. Agron. Sustain. Dev..

[9] Székács A., Darvas B., Ishaaya I., Palli S.R., Horowitz A.R. (2012). Comparative aspects of Cry toxin usage in insect control. Advanced Technologies for Managing Insect Pests.

[10] EPA (U.S. Environmental Protection Agency) (2010). Cry1Ab and Cry1F Bacillus Thuringiensis (Bt) Corn Plant-Incorporated Protectants.

[11] EPA (U.S. Environmental Protection Agency) (2010). Bacillus Thuringiensis Cry34Ab1 and Cry35Ab1 Proteins and the Genetic Material Necessary for Their Production (PHP17662 T-DNA) in Event DAS-59122–7 Corn (OECD Unique Identifier: DAS-59122–7).

[12] Darvas B., Bánáti H., Takács E., Lauber É., Szécsi Á., Székács A. (2011). Relationships of *Helicoverpa armigera*, *Ostrinia nubilalis* and *Fusarium verticillioides* on *MON 810* maize. Insects.

[13] Ostry V., Ovesna J., Skarkova J., Pouchova V., Ruprich J. (2010). A review on comparative data concerning *Fusarium* mycotoxins in *Bt* maize and non-*Bt* isogenic maize. Mycotoxin Res..

[14] Bowers E., Hellmich R., Munkvold G. (2014). Comparison of *Fumonisin contamination* using HPLC and ELISA methods in *Bt* and near-isogenic maize hybrids infested with European corn borer or Western bean cutworm. J. Agric. Food Chem..

[15] Spanjer M.C., Rensen P.M., Scholten J.M. (2008). LC-MS/MS multi-method for mycotoxins after single extraction, with validation data for peanut, pistachio, wheat, maize, cornflakes, raisins and figs. Food Addit. Contam. Part A Chem. Anal. Control Expo. Risk Assess..

[16] Rasmussen R.R., Storm I.M.L.D., Rasmussen P.H., Smedsgaard J., Nielsen K.F. (2010). Multi-mycotoxin analysis of maize silage by LC-MS/MS. Anal. Bioanal. Chem..

[17] Bryla M., Jedrzejczak R., Szymczyk K., Roszko M., Obiedziski M.W. (2014). An LC-IT-MS/MS-based method to determine trichothecenes in grain products. Food Anal. Methods.

[18] Hickert S., Gerding J., Ncube E., Hübner F., Flett B., Cramer B., Humpf H.U. (2015). A new approach using micro HPLC-MS/MS for multi-mycotoxin analysis in maize samples. Mycotoxin Res..

[19] Streit E., Schwab C., Sulyok M., Naehrer K., Krska R., Schatzmayr G. (2013). Multi-mycotoxin screening reveals the occurrence of 139 different secondary metabolites in feed and feed ingredients. Toxins.

[20] Székács A. (1998). Enzyme-linked immunosorbent assay for monitoring the *Fusarium* toxin zearalenone. Food Technol. Biotechnol..

[21] Wang S., Quan Y., Lee N., Kennedy I.R. (2006). Rapid determination of fumonisin B1 in food samples by enzyme-linked immunosorbent assay and colloidal gold immunoassay. J. Agric. Food Chem..

[22] Zhang A., Ma Y., Feng L., Wang Y., He C., Wang X., Zhang H. (2011). Development of a sensitive competitive indirect ELISA method for determination of ochratoxin A levels in cereals originating from Nanjing, China. Food Control.

[23] Sapsford K.E., Taitt C.R., Fertig S., Moore M.H., Lassman M.E., Maragos C.M., Shriver-Lake L.C. (2006). Indirect competitive immunoassay for detection of aflatoxin B1 in corn and nut products using the array biosensor. Biosens. Bioelectron..

[24] Adányi N., Levkovets I.A., Rodriguez-Gil S., Ronald A., Váradi M., Szendrő I. (2007). Development of immunosensor based on OWLS technique for determining aflatoxin B1 and ochratoxin A. Biosens. Bioelectron..

[25] Székács A., Adányi N., Székács I., Majer-Baranyi K., Szendrő I. (2009). Optical waveguide light-mode spectroscopy immunosensors for environmental monitoring. Appl. Opt..

[26] Majer-Baranyi K., Székács A., Szendrő I., Kiss A., Adányi N. (2011). Optical waveguide lightmode spectroscopy technique-based immunosensor development for deoxynivalenol determination in wheat samples. Eur. Food Res. Technol..

[27] Tittlemier S.A., Roscoe M., Drul D., Blagden R., Kobialka C., Chan J., Gaba D. (2013). Single laboratory evaluation of a planar waveguide-based system for a simple simultaneous analysis of four mycotoxins in wheat. Mycotoxin Res..

[28] Novo P., Moulas G., Prazeres D.M.F., Chu V., Conde J.P. (2013). Detection of ochratoxin A in wine and beer by chemiluminescence-based ELISA in microfluidics with integrated photodiodes. Sens. Actuators B Chem..

[29] Anderson G.P., Kowtha V.A., Taitt C.R. (2010). Detection of fumonisin B1 and ochratoxin A in grain products using microsphere-based fluid array immunoassays. Toxins.

[30] Aqai P., Peters J., Gerssen A., Haasnoot W., Nielen M.W.F. (2011). Immunomagnetic microbeads for screening with flow cytometry and identification with nano-liquid chromatography mass spectrometry of ochratoxins in wheat and cereal. Anal. Bioanal. Chem..

[31] Peters J., Bienenmann-Ploum M., De Rijk T., Haasnoot W. (2011). Development of a multiplex flow cytometric microsphere immunoassay for mycotoxins and evaluation of its application in feed. Mycotoxin Res..

[32] Czéh A., Mándy F., Fehér-Tóth S., Török L., Mike Z., Kőszegi B., Lustyik G. (2012). A flow cytometry based competitive fluorescent microsphere immunoassay (CFIA) system for detecting up to six mycotoxins. J. Immunol. Methods.

[33] Nolan J.P., Mándy F. (2006). Multiplexed and microparticle-based analyses: Quantitative tools for the large-scale analysis of biological systems. Cytometry Part A.

[34] Bashashati A., Brinkman R.R. (2009). A survey of flow cytometry data analysis methods. Adv. Bioinform..

[35] Goertz A., Zuehlke S., Spiteller M., Steiner U., Dehne H.W., Waalwijk C., de Vries I., Oerke E.C. (2010). *Fusarium* species and mycotoxin profiles on commercial maize hybrids in Germany. Eur. J. Plant Pathol..

[36] Catangui M.A., Berg R.K. (2006). Western bean cutworm, *Striacosta albicosta* (Smith) (Lepidoptera: Noctuidae), as a potential pest of transgenic Cry1Ab *Bacillus thuringiensis* corn hybrids in South Dakota. Environ. Entomol..

[37] Folcher L., Jarry M., Weissenberger A., Gérault F., Eychenne N., Delos M., Regnault-Roger C. (2009). Comparative activity of agrochemical treatments on mycotoxin levels with regard to corn borers and *Fusarium* mycoflora in maize (*Zea mays* L.) fields. Crop Prot..

[38] Schollenberger M., Müller H.-M., Ernst K., Sondermann S., Liebscher M., Schlecker C., Wischer G., Drochner W., Hartung K., Piepho H.-P. (2012). Occurrence and distribution of 13 trichothecene toxins in naturally contaminated maize plants in Germany. Toxins.

[39] Bayman P., Baker J.L., Doster M.A., Michailides T.J., Mahoney N.E. (2002). Ochratoxin production by the *Aspergillus ochraceus* group and *Aspergillus alliaceus*. Appl. Environ. Microbiol..

[40] Frisvad J.C., Skouboe P., Samson R.A. (2005). Taxonomic comparison of three different groups of aflatoxin producers and a new efficient producer of aflatoxin B1, sterigmatocystin and 3-*O*-methylsterigmatocystin, *Aspergillus rambellii* sp. nov.. Syst. Appl. Microbiol..

[41] Székács A., Darvas B. (2012). Environmental assessment of MON 810 maize in the *Pannonian biogeographical* region. Acta Phytopathol. Entomol. Hung..

[42] Seres A., Kiss I., Nagy P., Sály P., Darvas B., Bakonyi G. (2014). Arbuscular mycorrhizal fungi colonisation of Cry3 toxin-producing *Bt* maize and near isogenic maize. Plant Soil Environ..

[43] Icoz I., Stotzky G. (2008). Fate and effects of insect-resistant *Bt* crops in soil ecosystems. Soil Biol. Biochem..

[44] Balmas V., Scherm B., Marcello A., Beyer M., Hoffmann L., Migheli Q., Pasquali M. (2015). *Fusarium* species and chemotypes associated with *Fusarium* head blight and *Fusarium* root rot on wheat in Sardinia. Plant Pathol..

[45] Application Protocol for Fungi-Plex™ Kit. http://softflow.hu/Download/docs/fungi/Fungi_Plex_6_TDS_Application_Protocol%202012_05.pdf.

